# Sex Differences in Autism Spectrum Disorder: Repetitive Behaviors and Adaptive Functioning

**DOI:** 10.3390/children8050325

**Published:** 2021-04-22

**Authors:** Martina Siracusano, Valentina Postorino, Assia Riccioni, Leonardo Emberti Gialloreti, Monica Terribili, Paolo Curatolo, Luigi Mazzone

**Affiliations:** 1 Department of Biomedicine and Prevention, University of Rome Tor Vergata, Via Montpellier 1, 00133 Rome, Italy; leonardo.emberti.gialloreti@uniroma2.it; 2 Department of Biotechnological and Applied Clinical Sciences, University of L’Aquila, Via Vetoio 40, 67100 L’Aquila, Italy; 3 Department of Pediatrics, School of Medicine, Anschutz Medical Campus, University of Colorado, JFK Partners, Education 2 South (L28), 13121 E. 17th Ave., Aurora, CO 80045, USA; valentina.postorino86@gmail.com; 4 Child Neurology and Psychiatry Unit, Systems Medicine Department, University of Rome Tor Vergata, Via Montpellier 1, 00133 Rome, Italy; assiariccioni@gmail.com (A.R.); monica.terribili@gmail.com (M.T.); curatolo@uniroma2.it (P.C.); luigi.mazzone@uniroma2.it (L.M.)

**Keywords:** repetitive and restricted behaviors, sex differences, adaptive functioning, autism spectrum disorder

## Abstract

Sex differences in restricted and repetitive behaviors (RRBs) in individuals with Autism Spectrum Disorder (ASD) have been explored with mixed findings. We aimed to investigate sex differences in RRBs through a specific measure—i.e., the Repetitive Behavior Scale Revised (RBS-R)—in a sample of preschool-age and school-age children with ASD. Additionally, we evaluated if RRBs were differently related to adaptive functioning within the male and the female age groups. A sample of 210 ASD individuals (3–18 years; 145 males, 65 females) underwent an in-depth assessment including a cognitive, adaptive functioning evaluation (i.e., the Adaptive Behavior Assessment System, Second Edition (ABAS-II)) and RRBs assessment (i.e., RBS-R). No significant sex differences on the RBS-R total score or any RBS-R subscale emerged. Within the group of older participants, RRBs were negatively associated with all adaptive skill domains independently from sex and age. Our results suggest a lack of sex differences in RRBs in our sample. Additionally, our findings highlight the possible negative impact of RRBs on adaptive skills in older individuals with autism, emphasizing the need for autistic individuals of both sexes to undergo an early intervention targeting RRBs, in order to improve their adaptive skills.

## 1. Introduction

Autism Spectrum Disorder (ASD) is an early-onset and lifelong neurodevelopmental condition characterized by social communication and social skill deficits, restricted interests, and repetitive behaviors (Diagnostic and Statistical Manual of Mental Disorders, Fifth Edition (DSM-5)) [1]. Recent data report a 4.3:1 male to female ratio for this disorder, with a higher percentage of females meeting criteria for intellectual disability [2]. A growing body of research has investigated sex differences in phenotypic presentation in individuals with ASD and the findings of these studies are mixed [3,4,5,6,7,8,9,10,11,12,13,14,15,16,17,18,19].

The majority of studies have described lower cognitive and language skills [3,4,5,6,7], greater impairment in social communication skills [5,6,7,8,9], fewer restricted and repetitive behaviors (RRBs) [4,7,9,10,11], lower adaptive skills [5,6], and greater problem behaviors [6,7] in females with ASD.

On the other hand, other studies have reported that autistic females show less verbal and nonverbal communicative difficulties [12], no differences in adaptive skills [13,14,15,16], greater interest in social relationships and friendships, better imagination skills, and fewer, or at least less, atypical RRBs compared to males [12,17,18,19]. It is worth noting that these sex differences in symptoms presentation may represent one of the reasons why autism can be misdiagnosed or underdiagnosed in females [20]. 

Over the past few years, studies have increasingly focused on investigating sex differences in RRBs in this clinical population. RRBs specifically include a broad category of behaviors: restricted interests (atypical for content and intensity), repetitive use of the objects, stereotyped language, repetitive motor mannerisms, insistence on sameness, unusual sensory behaviors, and strict adherence to non-functional routines or rituals [1]. Available findings on sex difference in RRBs are inconsistent. In fact, some studies reported fewer RRBs in females [6,8,9,10,11,12,17,18,19,21,22,23,24,25], whereas others did not find significant sex differences [15,26,27,28,29,30,31].

These conflicting results may be due to differences in sample size (e.g., small female sample size), ascertainment of the study population, and methods of evaluation. To date, few studies have used specific measures (e.g., Repetitive Behavior Scale Revised (RBS-R)) to assess sex differences in RRBs in this population [6,22,27,29,30,31]. The majority of the studies have investigated RRBs through instruments that do not give an extensive evaluation of these behaviors, such as direct observation, the Autism Diagnostic Observation Schedule Generic (ADOS-G) [32] and the ADOS Second Edition (ADOS-2) [33] RRB domain, and the Autism Diagnostic Interview—Revised RRB items or domain [6,9,10,12,17,18,19,23,25,26,28,34,35,36]. For example, Knutsen et al. [28] did not find any sex difference in RRBs through the use of the ADOS-2 in a sample of 1024 ASD children selected from the Autism Treatment Network (ATN) registry. It has to be noted that these authors identified as the main limit of their study the employment of the ADOS-2 to assess RRBs instead of a specific measure (e.g., the Repetitive Behavior Scale Revised (RBS-R) or the Repetitive Behavior Scale for Early Childhood (RBS-EC) [37,38]. In particular, the authors stated that ADOS-2 “does not provide a comprehensive assessment of RRBs” [28]. Moreover, given that RRBs are symptoms that may appear just in certain conditions (i.e., excitement, agitation, frustration, fear), they can be hardly captured by the administration of the ADOS-2 which occurs in a restricted time and in a specific setting [39].

Moreover, in order to characterize the sex differences in the phenotypic presentation of ASD, a number of studies investigated the level of adaptive behavior between the different sexes in the autistic population without consistent results [5,6,15,25,40]. For example, Maravic et al. [15] reported similar results in both sexes within a sample of 108 individuals with ASD, even if a trend of better functioning characterized females. On the contrary, in a study of Ratto et al. [25], a significant impairment in daily living skills was reported by parents of females with ASD. Even in this case, the inconsistent findings have been addressed to methodological issues such as different inclusion criteria of the samples (i.e., age, co-occurring conditions).

Regarding the relationship between RRBs and adaptive functioning in ASD, several studies have shown that the presence of RRBs can have a negative impact on the level of functioning in this clinical population [10,40,41,42,43,44,45,46,47,48]. These studies have been driven by the hypothesis that the presence of RRBs affects the individual’s ability to maintain attention on activities that promote development, thus leading to a delay in different skills (e.g., social, communication, cognitive). Furthermore, there is evidence that the presence of RRBs during preschool years predicts poorer adaptive abilities in later childhood [48]. To our knowledge, most of the studies investigating sex differences in this clinical population have not specifically focused on the relationship between adaptive skills and RRBs, but they have generally investigated the relation between adaptive functioning and ASD core symptoms (i.e., social communication and social skill deficits, restricted interests, and repetitive behaviors), including RRBs in this broad category of symptoms [15,49].

The aim of the current study was to investigate sex differences in RRBs through a specific measure (i.e., RBS-R) in a sample of preschool-age and school-age children with autism. Additionally, we examined sex differences in the relationship between RRBs and adaptive functioning within the two age groups (preschool-age and school-age).

We hypothesized a different profile of repetitive behaviors across sexes and we expected that repetitive behaviors would be negatively related to the adaptive functioning.

## 2. Materials and Methods

### 2.1. Participants

A total of 210 participants with ASD (age range 3–18 years), of which 58 preschoolers (0–5 years) and 152 school-age individuals (>6 years), were included in the study. Of these, 145 were males (mean age: 9.1 ± 4.13 years) and 65 were females (mean age: 8.1 ± 4.36 years). In particular, within the preschooler group, 34 were males and 24 females; within school-age group, there were 111 males and 41 females. All participants were recruited throughout the Child Psychiatry Unit of the University Hospital Tor Vergata of Rome (Italy).

Participants underwent a medical and developmental assessment, including a diagnostic evaluation (see paragraph below) performed by a multidisciplinary team (e.g., child psychiatrists, clinical psychologists). ASD had to be diagnosed according to the Diagnostic and Statistical Manual of Mental Disorders-Fifth Edition (DSM-5) [1]. The Autism Diagnostic Observation Schedule, Second Edition (ADOS-2) [33] was administered by a trained licensed clinician and employed to support the clinical diagnosis of autism spectrum disorder. Exclusion criteria for all participants included genetic disorders, epilepsy, or other medical disorders.

### 2.2. Instruments

#### 2.2.1. ASD Diagnostic Measure

Autism Diagnostic Observation Schedule, Second Edition (ADOS-2) [33].

All participants were assessed for the presence of autism symptoms—including socio-communicative difficulties, and repetitive and restricted behaviors—through the ADOS-2, a semi-structured observational assessment, performed by a licensed trained clinician in order to support the clinical diagnosis of ASD. The ADOS-2 is divided into modules. The choice of modules is based on the participant’s age and expressive language level. In the present study, the participants were administered different modules according to their age and expressive language level. The ADOS-2 provides a Calibrated Severity Score (CSS) which permits to compare scores across modules.

#### 2.2.2. Cognitive and Adaptive Functioning Measures

All participants underwent a cognitive and adaptive evaluation. To assess participants’ intellectual quotient (IQ), we used either the Leiter International Performance Scale-Revised [50], the Wechsler Preschool and Primary Scale of Intelligence-Third Edition [51], or the Wechsler Intelligence Scale for Children–Fourth Edition (WISC-IV) [52]. The choice of the cognitive measure was based on age, expressive language level, and the ability to engage and cooperate of each participant. All of these measures used the same standard scores (SS = 100) and standard deviations (SD = 15).

Furthermore, all participants’ parents completed the Adaptive Behavior Assessment System, Second Edition (ABAS-II) in order to assess their child’s adaptive functioning [53].

The ABAS-II is a parent-report questionnaire which measures child’s skills related to development, behavior, and cognitive abilities. Participants’ caregivers were administered the “0–5 years” form or the “5–21 years” form according to their child’s age.

Parents are asked to rate the child’s skills to complete an activity (from 0 = “not able to” to 3 = “able to do it and always performs it when needed”) in regards to 10 functioning areas (i.e., communication, use of environment, preschool competences, domestic behavior, health and safety, play, self-care, self-control, social abilities, and motility) gathered in three main adaptive domains: conceptual (CAD), practical (PAD), social (SAD), and a comprehensive score, General Adaptive Composite (GAC), given by the sum of scaled scores from the 10 skill areas.

Composite scores (M 100± SD 15) of all adaptive domains (CAD, PAD, SAD, GAC) were used for the analysis and were analyzed according to the ABAS-II form that was administered (i.e., “0–5 years” and “5–21 years”).

#### 2.2.3. Repetitive Behavior and Restricted Interests Assessment

Repetitive behavior and restricted interests were assessed through the Italian version [29] of the Repetitive Behavior Scale Revised (RBS-R), a parent-report checklist [54]. The RBS-R includes 43 items rated on a 4-point Likert scale. Items are organized in six subscales: (1) Stereotypic Behavior, (2) Self-injurious Behavior, (3) Compulsive Behavior, (4) Ritualistic Behavior, (5) Sameness Behavior, and (6) Restricted Interests Behaviors. 

We used a five-factor solution scoring [38] which consists of the integration of two subscales (Ritualistic Behavior and Sameness Behavior) into one (i.e., the Ritualistic/Sameness Behavior).

The raw score of each subscale was calculated in addition to an RBS Total score, a comprehensive sum of all the subscales’ raw score.

### 2.3. Statistical Analysis

Independent sample *t*-tests were performed to evaluate sex differences in the demographic variables (i.e., age, intelligence quotient (IQ), and autism severity level) (Table 1), RRBs, and adaptive functioning (Table 2). Spearman’s correlations were used to evaluate the relationship between RRBs and adaptive functioning. To further explore associations among these variables, multiple regression analyses were used (Table 3). In a hierarchical multiple linear regression model, the RBS-R Total score was used as dependent variable, and the IQ, age, and sex were entered as independent variables in three different steps. Before performing the hierarchical multiple regression analysis, the independent variables were examined for collinearity. The results of the variance inflation factor (all less than 2.0) and collinearity tolerance (all greater than 0.76) suggested that the estimated Beta coefficients were well established. Other multiple regression models were used where the different subscales of the ABAS-II were entered as dependent variables, and the IQ, age, RBS-R Total score, and sex were entered as independent variables (Table 3). For all multiple regression analyses, the dummy variable sex was coded as 0 = male and 1 = female.

General linear models (GLM) with tests between-subjects effects with ABAS-II subscales (GAC, CAD, SAD, PAD) as dependent variable were used to test for possible interactions between explanatory variables, such as age group (preschooler/schooler), sex (male/female), and RBS-R Total score.

An alpha level of 0.05 was used for all statistical analyses. When performing multiple comparisons (up to 16), we adjusted the *p*-value using the Bonferroni correction. To keep the family-wise error rate at <0.05, the alpha level was set at 0.003 for each comparison. The results are reported as means ± SDs if not otherwise specified. All analyses were performed using the Statistical Package for Social Sciences (SPSS) software (Version 25, Inc., Chicago, IL, USA).

## 3. Results

### 3.1. Sex Differences in Demographic Variables

Sex differences in demographic variables are reported in Table 1. No significant sex difference emerged concerning age (females: 8.1 ± 4.36 years; males: 9.1 ± 4.13 years *p* = 0.101), IQ (females: 87.2 ± 27.52; males: 86.1 ± 24.12, *p* = 0.814), and ADOS-2 severity level (females CSS: 6.31 ± 1.54; males CSS: 6.93 ± 1.9; *p* = 0.036).

### 3.2. Sex Differences in Repetitive Behaviors and Relationship with Age and Cognitive Functioning

No significant sex difference was found on the RBS-R Total score or any RBS-R subscale within both age groups, preschoolers and school-age children (Table 2). No significant correlation emerged between age and the RBS-R Total score within both sexes. The IQ was negatively correlated with the RBS-R Total score (*r =* −0.370 *p* < 0.001) only in the male group.

A multiple linear regression was calculated to evaluate the relation between the RBS-R Total score and sex, while adjusting for the participants’ IQ and age. A significant regression equation was found (F (3,136) =6.167, *p* = 0.001), with an R^2^ of 0.120. However, only the IQ was a significant negative predictor of the RBS-R Total score (Beta= −0.21; *p* < 0.001), while sex (Beta= −2.40; *p* = 0.42) and age (Beta = 0.31; *p* = 0.32) did not reach statistical significance. 

### 3.3. Sex Differences in Adaptive Functioning and Relationship with Repetitive Behaviors

No significant sex difference emerged on any ABAS-II domain in both age groups (“0–5 years” form and “5–21 years” form) (Table 2). 

Among school-age male children, we observed statistically significant negative correlations between RBS-R total score and all adaptive domains (GAC: R −0.490 *p* < 0.001; CAD R −0.364 *p* < 0.001; SAD R −0.431 *p* < 0.001; PAD R −0.457 *p* < 0.001). Whereas, amongst female schoolers, no statistically significant correlation was found between RBS-R Total score and any ABAS-II domain (GAC R −0.443 *p* 0.014; CAD R −0.342 *p* 0.069; SAD R −0.474 *p* 0.009; PAD R −0.423 *p* 0.020).

Among the preschooler group, no statistically significant correlation emerged, in both sexes, between all adaptive domains and the RBS-R Total score (male GAC R −0.531 *p* 0.013; female GAC R −0.064 *p* 0.0822).

Several multiple linear regressions were performed to explore the relation between the ABAS-II scores and the RBS-R Total score, while adjusting for participants’ sex, IQ, and age in the preschooler group, and for participants’ sex and IQ in the school-age group (Table 3). The composites scores of the three ABAS-II adaptive domains (i.e., CAD, PAD, and SAD) and the GAC were used as the dependent variables of four different models. A detailed description of the four multiple linear regressions models is presented in Table 3.

No statistically significant regression equation was found in relation to the “0–5 years” form (Table 3).

Statistically significant regression equations were attained for all the three ABAS-II domains and the GAC in relation to the “5–21 years” form (Table 3). A significant regression equation was found (F(4,97) = 19.129, *p* < 0.001), with an R^2^ of 0.441 when considering the GAC as a dependent variable. The RBS-R Total score (Beta = −0.28; *p* < 0.001) as well as the IQ (Beta = 0.28; *p* < 0.001) were significant predictors of the GAC score, while sex and age did not reach statistical significance in this age group. Specifically, the GAC showed a positive relation with the IQ and a negative one with the RBS-R Total score.

Similar results were obtained when considering the PAD domain; whereas IQ was a significant positive predictor of the CAD score (Beta = 0.31; *p* < 0.001), RBS-R Total score was negatively related to the SAD domain (Beta = −0.28; *p* < 0.001) (Table 3).

To evaluate the possible interactions between the explanatory variables with regards to the ABAS-II subscales, we performed four general linear models, with GAC, CAD, SAD, and PAD, respectively, as the dependent variable. Age group (preschooler/schooler), sex (male/female), and the continuous variable RBS-R total score were included as explanatory variables. No significant interaction between explanatory variables was found in any of the four models—only the main effect of the RBS-R total score was always statistically significant. For GAC as a dependent variable, the *p*-value was = 0.001 (F(1.143) = 12.53; Partial Eta Squared = 0.084).

## 4. Discussion

The aim of this study was to investigate sex differences in repetitive behaviors measured by a specific instrument (i.e., RBS-R) in a sample of preschooler and school-age children with autism. Additionally, we evaluated if RRBs differently affected the adaptive functioning within sexes in the two age groups (preschool-age, school-age). In line with previous studies, we found no sex differences in our sample on the RBS-R total score [6,30,31]. We found that in our sample, males and females were also similar on all the RBS-R subscales, which is in contrast with previous literature findings supporting that females show lower restricted behaviors compared to males [6,31].

Frazier et al. [6], analyzing sex differences in cognitive and behavioral characteristics in 2,418 individuals with ASD from the Simons Simplex Collection, found no sex differences in the RBS-R total score. By contrast, these authors found significant sex differences in the RBS-R restricted interests subscale and the ADI-R repetitive domain score, with females showing significantly lower repetitive behaviors [6]. Similarly, Fayden et al. [31], examining sex differences within restricted interests in a sample of 125 participants with and without ASD, found no sex differences in RRB severity, except for a lower score on the RBS-R Restricted Behavior subscale in female participants. However, it has to be noted that this study used for the analysis a RRB severity composite score generated using different parent-and clinician-report measures (i.e., the ADI-R RRBs subscale, the ADOS-2 RRBs subscale, the RBS-R total score, and the SRS-2 RRBs subscale) and only one subscale (i.e., the RBS-R restricted interest) was used independently as a measure of repetitive behaviors. Therefore, it is possible that differences in inclusion criteria and sample size (e.g., the study of Fayden et al. [31] included only 75 participants with ASD, a small female sample size (*n* = 20), and a wide age range (from 2–57 years)) have contributed to these differences in the RBS-R restricted interest subscale results. Furthermore, it has to be noted that females overall scored lower on this RBS-R subscale compared to males, even if this difference was not statistically significant.

Moreover, in a recent study of Antezana et al. [30] on 615 youth with ASD, even if gender differences did not emerge in the RBS-Total, the authors found that female participants were characterized by higher scores on specific RBS items (compulsive, insistence on sameness, restricted and self-injurious behavior) in comparison to males [30]. Interestingly, Antezana et al. underscored the need to understand whether the high rates of RRBs that emerged within females could be attributable to comorbid disorders, such as anxiety, rather than being a characteristic of the phenotypic presentation.

Notably, in our sample, IQ emerged as the only variable influencing the level of repetitive behaviors. Specifically, a higher IQ corresponded to a reduction of RRBs independently from age and sex. This is consistent with the literature reporting more repetitive behaviors in ASD individuals with lower cognitive level [44,54,55,56,57]. These results are also in line with the hypothesis that repetitive behaviors may be covered in higher functioning individuals with autism, and especially in females [6,20,58]. Specifically, it can be possible that girls may require a major etiological load to manifest an autistic phenotype, with the result that they may be undiagnosed or misdiagnosed—especially girls with an average or above average cognitive skill [6,58,59]. This hypothesis would also explain the higher unevenness in male to female ratios (10:1) in the ASD population when the cognitive functioning is considered [2].

Moreover, having female RRBs usually a less atypical content (animals like horses, fashion, popstars) in comparison to males (objects, numbers, letters) [20], these behaviors could be barely captured by a parental report measure (because not considered as significantly peculiar), contributing to the difficulty in the definition of a clearly behavioral phenotype across sexes. In relation to this, noteworthy is our finding that—even if no significant sex differences were found in RRBs within both age groups—interestingly, within preschoolers, males were characterized by higher scores in comparison to females. Whereas, within the school-age group, RBS-R scores were similar among male and female participants. These results suggest that females could later develop clear RRBs, or these behaviors may be less evident—and hardly captured by a parental report—at younger age and worsen overtime. How RRBs change overtime in ASD, and even more across sexes, represents a field of particular interest which necessitates further longitudinal studies [27,60].

Regarding the relationship between repetitive behaviors and adaptive functioning, our results showed that repetitive behaviors negatively influenced all adaptive skill domains within the group of older participants independently from sex and age, whereas due to the limited sample size of preschool-age group, we cannot draw any conclusion regarding preschoolers.

Our result is in line with previous findings and can be suggestive of a greater effect of these behavioral patterns on everyday life, especially in older age [10,41,42,43,48,57]. In fact, adaptive skills such as communication abilities, social skills, self-care, household skills, environmental exploration, health, and safety become more important with age, and daily engagement in RRBs can highly impact them.

Our results contribute to clarify the relationship between RRBs and adaptive functioning and point out the need for individuals with ASD of both sexes of a prompt intervention on repetitive behaviors.

However, several limitations characterize our research. First, the younger group included in our study is smaller than the older group. Second, we used parent-report measures (i.e., the RBS-R and the ABAS-II) rather than the clinician’s observation to assess repetitive behaviors and adaptive functioning, thus their results could have been influenced by parental opinion. Moreover, the present study, being cross-sectional, did not longitudinally examine the RRBs’ developmental trajectory of participants from early childhood to schooler age (how RRBs change overtime within ASD individuals; what are the predictive factors?). Finally, by using the Bonferroni correction for multiple comparisons, we were intentionally conservative in order to minimize possible false positives. However, also considering the relatively small sample size and, therefore, the limited power of the study, we might have overlooked some actual associations. Further studies on wider samples are necessary in order to confirm and better clarify our findings.

## 5. Conclusions

This study examined sex differences in repetitive behaviors in a group of individuals with ASD. Furthermore, we investigated the relationship between repetitive behaviors and adaptive functioning within the male and the female group.

Our results suggest a lack of sex differences in repetitive behaviors, measured by RBS-R, in our sample. Additionally, our findings highlight the possible negative impact of RRBs on adaptive skills in individuals with ASD, regardless of sex. 

The results of our study point out the need to further investigate, specifically at early stages of development, repetitive behaviors (not including them in the broad category of ASD core symptoms) as possible predictors of adaptive functioning in ASD children. 

Subsequently, our findings emphasize the need for individuals with ASD of both sexes to undergo an early intervention targeting repetitive behaviors and adaptive skills.

Indeed, further longitudinal studies—on comparable group size of individuals with ASD belonging to preschooler and older ages—investigating sex differences in the relationship between repetitive behaviors and adaptive functioning are required in this clinical population in order to better clarify the long-term effect of these behaviors on subsequent outcomes.

## Figures and Tables

**Table 1:** Sex Differences in Demographic Variables.

	Females (*n* = 65) M ± SD	Males (*n* = 145) M ± SD	*t*	*p* Value
**Age (years)**	8.1 ± 4.36	9.1 ± 4.13	1.648	0.101
**IQ**	87.2 ± 27.52	86.1 ± 24.12	−0.236	0.814
**ADOS-2 CSS**	6.31 ± 1.54	6.93 ± 1.9	2.115	0.036

ADOS-2 CSS = Autism Diagnostic Observation Schedule, Second Edition calibrated severity score; IQ = intelligence quotient; M = mean; SD = standard deviation.

**Table 2:** Sex Differences in Adaptive Functioning and RRBs.

	Females	Males	*t*	*p* Value
	M ± SD	M ± SD		
**ABAS-II**				
Preschool-age group				
“0–5 years” form				
GAC	65.60 ± 14.29	59.43 ± 15.66	−1.208	0.236
CAD	68.47 ± 14.28	63.62 ± 14.67	−0.988	0.33
SAD	69.67 ± 14.71	65.29 ± 18.36	−0.764	0.45
PAD	69.93 ± 11.28	61.24 ± 15.17	−1.829	0.077
School-age group				
“5–21 years” form				
GAC	61.90 ± 17.30	58.83 ± 14.10	−0.966	0.336
CAD	66.93 ± 19.17	65.48 ± 14.38	−0.379	0.707
SAD	68.90 ± 14.81	65.90 ± 13.45	−1.016	0.312
PAD	63.23 ± 18.58	57.09 ± 16.55	−1.703	0.091
**RBS-R**				
Preschool-age group				
Stereotypic	5.46 ± 5.16	7.49 ± 4.51	1.59	0.116
Self-Injurious	0.71 ± 1.12	1.20 ± 1.53	1.34	0.184
Compulsive	1.38 ± 2.49	2.66 ± 3.50	1.54	0.129
Ritualistic/Sameness	4.38 ± 4.22	5.37 ± 5.54	0.74	0.46
Restricted Interests	1.83 ± 1.63	2.77 ± 2.30	1.72	0.091
Total	13.75 ± 11.08	19.49 ± 13.54	1.72	0.092
School-age group				
Stereotypic	5.68 ± 6.4	6.12 ± 5.12	0.43	0.666
Self-Injurious	1.95 ± 3.23	1.52 ± 2.62	−0.83	0.405
Compulsive	3.51 ± 3.96	3.17 ± 3.49	−0.51	0.612
Ritualistic/Sameness	8.59 ± 8.10	8.46 ± 7.11	−0.09	0.926
Restricted Interests	3.12 ± 2.46	3.36 ± 2.45	0.52	0.6
Total	23.53 ± 20.18	22.72 ± 15.78	−0.26	0.798

ABAS-II = Adaptive Behavior Assessment System, Second Edition; CAD = conceptual adaptive domain; GAC = General Adaptive Composite score; IQ = intelligence quotient; M = mean;; PAD = practical adaptive domain; RBS-R = Repetitive Behavior Scale-Revised; SAD = social adaptive domain; SD = standard deviation.

**Table 3:** Relationship between Adaptive Functioning and Repetitive Behaviors: multiple regression analysis.

Preschool-Age Group																				
	GAC ^1^					CAD ^2^					SAD ^3^					PAD ^4^				
	ß	SE	*t*	*p*	95%CI	ß	SE	*t*	*p*	95%CI	ß	SE	*t*	*p*	95%CI	ß	SE	*t*	*p*	95%CI
**I**	0.26	0.2	1.28	0.21	−0.16	0.37	0.18	2.02	0.05	−0.01	0.29	0.24	1.2	0.23	−0.2	0.21	0.18	1.17	0.25	−0.17
**Q**					0.69					0.75					0.79					0.6
**R**	−0.51	0.31	−1.64	0.11	−1.16	−0.43	0.27	−1.5	0.13	−1.0	−0.67	0.36	−1.8	0.07	−1.4	−0.62	0.28	−2.2	0.037	−1.20
**B**					0.13					0.14					0.08					-0.04
**S**																				
**S**	7.63	7.48	1.02	0.32	−7.97	2.42	6.58	0.36	0.71	−11.3	1.1	8.64	0.12	0.89	−16.9	4.51	6.69	0.67	0.5	−9.43
**E**					23.24					16.1					19.1					18.47
**X**																			
**School-Age Group**																				
	**GAC ^5^**					**CAD ^6^**					**SAD ^7^**					**PAD ^8^**				
	**ß**	**SE**	***t***	***p***	**95%CI**	**ß**	**SE**	***t***	***p***	**95%CI**	**ß**	**SE**	***t***	***p***	**95%CI**	**ß**	**SE**	***t***	***p***	**95%CI**
**A**	0.49	0.3	1.64	0.1	−0.1	0.17	0.34	0.49	0.62	−0.5	−0.07	0.31	−0.22	0.83	−0.69	0.73	0.38	1.93	0.06	−0.02
**G**					1.09					0.84					0.55					1.48
**E**																				
**I**	0.28	0.05	5.69	**<0.001**	0.18	0.31	0.05	5.65	**<0.001**	0.2	0.12	0.05	2.48	0.01	0.02	0.23	0.06	3.76	**<0.001**	0.1
**Q**					0.37					0.42					0.22					0.35
**R** **B** **S**	−0.28	0.07	−3.95	**<0.001**	−0.43	−0.21	0.08	−2.64	0.01	−0.37	−0.3	0.07	−4.03	**<0.001**	−0.45	−0.32	0.09	−3.57	**0.001**	−0.5
					−0.14					−0.05					−0.15					−0.14
**S**	2.6	2.55	1.02	0.31	−2.45	0.49	2.89	0.17	0.86	−5.23	1.86	2.68	0.69	0.49	−3.48	6.13	3.19	1.92	0.06	−0.19
**E** **X**					7.66					6.23					7.19					12.46

^1^ Overall model: F = 1.87; df: 23; *p* = 0.166; R^2^ = 0.219; ^2^ overall model: F = 1.85; df: 23; *p* = 0.171; R^2^ = 0.217; ^3^ overall model: F = 1.40; df: 23; *p* = 0.273; R^2^ = 0.173; ^4^ overall model: F = 2.31; df: 23; *p* = 0.107; R^2^ = 0.257. ^5^ overall model: F = 19.13; df: 101; *p* < 0.001; R^2^ = 0.441; ^6^ overall model: F = 13.85; df: 102; *p* < 0.001; R^2^ = 0.361; ^7^ overall model: F = 8.20; df: 102; *p* < 0.001; R^2^ = 0.251; ^8^ overall model: F = 12.28; df: 103; *p* < 0.001; R^2^ = 0.332. Significant values are reported in bold.

## Data Availability

The data presented in this study are contained within the article.

## Abbreviations

ASD	Autism Spectrum Disorder
IQ	Intelligence Quotient
RRBs	Restricted and Repetitive Behaviors
RBS-R	Repetitive Behavior Scale Revised
ABAS-II	Adaptive Behavior Assessment System, Second Edition
GAC	General Adaptive Composite
DAC	Conceptual Adaptive Domain
DAS	Social Adaptive Domain
DAP	Practical Adaptive Domain
ADOS-2	Autism Diagnostic Observation Schedule, Second Edition
ADOS-G	Autism Diagnostic Observation Schedule-Generic
ADI-R	Autism Diagnostic Interview Revised
ADHD	Attentions Deficit and Hyperactivity Disorder
